# Fingerprinting of Plumbagin in *lic>Drosera burmannii* Vahl using High Performance Thin Layer Chromatography

**DOI:** 10.4103/0250-474X.49127

**Published:** 2008

**Authors:** V. Madhavan, Hema Basnett, A. Cendil Kumar, S. N. Yoganarasimhan

**Affiliations:** Department of Pharmacognosy, M. S. Ramaiah College of Pharmacy, Bangalore-560 054, India; 1 Department of Pharmaceutical Chemistry, M. S. Ramaiah College of Pharmacy, Bangalore-560 054, India

**Keywords:** *Drosera burmannii*, HPTLC fingerprinting, marker component, plumbagin

## Abstract

HPTLC fingerprinting profile of the alcohol and aqueous extracts of *Drosera burmannii* is described. Seven components have been detected in the alcohol extract. Further, plumbagin, an useful antifertility agent, was also detected by comparison with the reference standard. The aqueous extract revealed two spots with no spot corresponding to plumbagin.

*Drosera burmannii* Vahl (Droseraceae) is an insectivorous, glandular, hairy herb, with rose coloured flowers occurring throughout India up to 2666 m^1^ and is reported to have rubefacient property^1^,^2^. It contains 1,4-naphthoquinones, plumbagin, ramantaceon and its glucoside rossoliside^3^, flavonoids like quercetin and hyperoside^4^. Plumbagin is 5-hydroxy-2-methyl-1,4-naphthoquinone, a yellow colour pigment found in Plumbaginaceae and Droseraceae^5^,^6^. Plumbagin possesses antifertility^7^, antimalarial^8^, antiviral^9^, antimicrobial^10^, anticancer^11^ and leishmanicidal^12^ activity. Different species of *Drosera* L. like *D. rotundifolia* L. are used in whooping cough, fever, mental and stomach disorders, skin diseases in homeopathic system of medicine^2^. The objective of the present study was to develop HPTLC-aided fingerprint profile of *D. burmannii*, which may be used as markers for quality evaluation, and standardization of the drug.

*D. burmannii* was collected from forests of Savanadurga, Bangalore during February 2006 and was authenticated. A voucher herbarium specimen (*Hema Basnett* 005) along with a voucher drug sample is preserved at this College herbarium and crude drug museum. The material was washed, shade dried, powdered, passed through sieve no. 60 and stored in airtight containers in day light for three months at room temperature (±20°). Plumbagin reference standard was procured from HiMedia, Mumbai. The air dried powder was successively extracted with 95% ethanol in a Soxhlet apparatus and finally the marc was macerated with chloroform water (0.25%) for 24 h to obtain the aqueous extract. The extracts were further concentrated under vacuum using a rotary flash evaporator and dried in a desiccator.

Camag HPTLC system equipped with Linomat V sample applicator, Camag TLC Scanner 3 and WinCATS 4 software for interpretation of the data was used. An aluminium plate (20×10 cm) precoated with silica gel 60F_254_ (E. Merck) was used as adsorbent. The plates were developed using toluene:glacial acetic acid (55:1) and toluene:chloroform:glacial acetic acid (1:1:0.1) as mobile phase for alcohol and aqueous extracts respectively in a Camag twin trough chamber to a distance of 8 cm each.

Solution of plumbagin reference standard (1 mg/ml) was prepared in alcohol as stock solution. Solution of the alcohol extract was prepared by dissolving the extract in alcohol. The aqueous extract was dissolved in alcohol, filtered, the filtrate used as aqueous extract solution. The TLC plates were activated by heating at 115° for about 30 min prior to use. The standard plumbagin solution and alcohol extract solution or aqueous extract solution were applied as 6 mm bands on two different precoated silica gel 60 F_254_ TLC plates, and the plates were developed in appropriate mobile phase. No prewashing of plates was carried out. Chamber saturation time was maintained at 1 h. The developed plates were allowed to dry and scanned at a wavelength of 425 nm, slit dimension 6.00×3.00 nm, scanning speed 20 nm/sec and the source of radiation was tungsten lamp. The R_f_ and peak area of the standard and the extracts were interpreted by using the software. The developed plates were photo documented using Camag Reprostar-3, equipped with a 12bit CCD camera, under 254, 366 nm and white light.

Plumbagin reference standard shows an R_f_ of 0.56 (fig. 1) and 0.66 in the mobile phase adopted for alcohol and aqueous extracts respectively. HPTLC analysis of alcohol and aqueous extracts of *D. burmannii* revealed different chromatographic profiles. In this study the alcohol extract revealed seven components at R_f_ 0.12, 0.18, 0.21, 0.24, 0.29, 0.56, 0.81 (figs. 2 and 3). Out of these, the most pronounced spot of maximum area was at R_f_ 0.56, corresponding to that of marker compound plumbagin. Three other spots at R_f_ 0.12, 0.18 and 0.21 were also prominent.

**Fig. 1:**
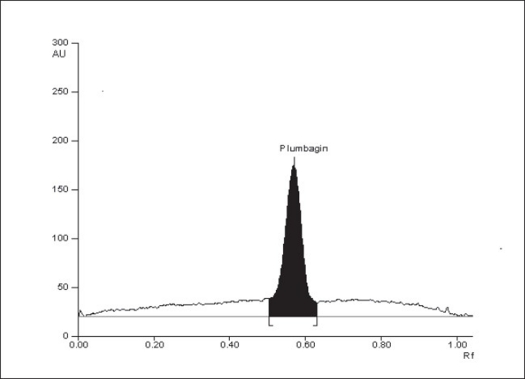
HPTLC chromatogram of plumbagin HPTLC chromatogram of a standard solution of plumbagin at 425 nm

**Fig. 2:**
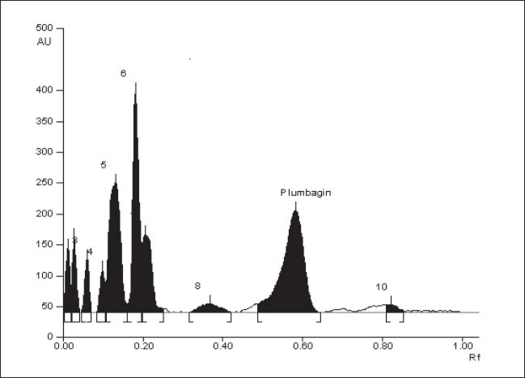
HPTLC chromatogram of alcohol extract HPTLC chromatogram of alcohol extract of *Drosera burmannii* at 425 nm

**Fig. 3:**
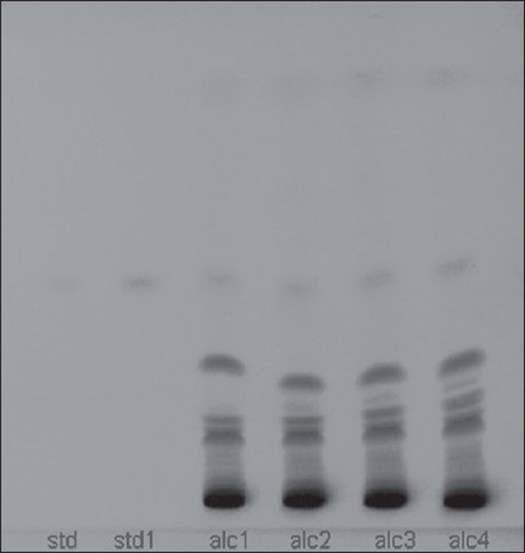
Chromatogram of alcohol extract Std, std1 is the plumbagin standard (254 nm) alc1, alc2, alc 3 and alc 4 are alcohol extract.

The method is specific for plumbagin in alcohol extract since it resolves the peak of plumbagin in the mobile phase proposed for the alcohol extracts to an R_f_ of 0.56 in the presence of other components. The specificity was confirmed by overlaying the spectra of plumbagin in reference standard (λ_max_ 425 nm), with the absorption spectrum obtained from the corresponding band in the track of alcohol extract (fig. 4). The aqueous extract revealed spots at R_f_ 0.15 and 0.24 with no spot corresponding to that of plumbagin reference standard (R_f_ 0.66). This is due to the hydrophobic nature of plumbagin^13^. The study is the first report on HPTLC profile of *D. burmannii*, which reveals components useful for quality evaluation, and standardization of the drug. Further it confirms the presence of plumbagin as marker compound in *D. burmannii*.

**Fig. 4:**
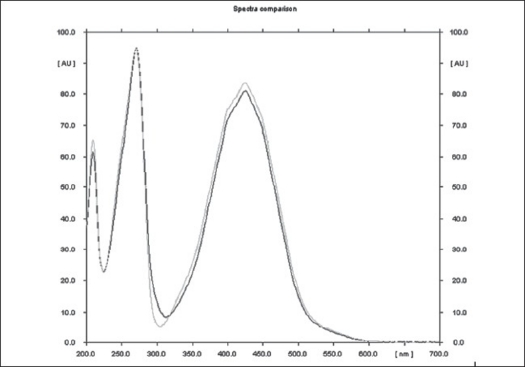
Overlay spectrum of plumbagin and alcohol extract Overlay spectrum of plumbagin and alcohol extract of *Drosera burmannii* at a λmax 425 nm

## References

[1] Santapau H, Henry AN (1976). A Dictionary of the Flowering Plants in India.

[2] Anonymous (1989). The Wealth of India: A Dictionary of Indian Raw Materials and Industrial Products.

[3] Wagner H, Baldt S, Zgainski EM (1984). Plant Drug Analysis.

[4] Wang Q, Su J, Zeng (1998). The isolation and identification of flavonoids from *Drosera burmanni*. Zhong Yao Cai.

[5] Botanical Dermatology Database.

[6] http://bodd.cf.ac.uk/BotDermFolder/BotDermP/DROS.html.

[7] Premakumari P, Rathinam K, Santhakumari (1977). Antifertility activity of plumbagin. Indian J Med Res.

[8] Suraveratum N, Krungkrai SR, Leangaramgul P, Prapunwattana P, Krugkai J (2000). Purification and characterization of *Plasmodium falciparum* succinate dehydrogenase. Mol Biochem Parasitol.

[9] Min BS, Kim YH, Toomiyama M, Nakamura N, Miyashiro H, Otake T (2001). Inhibitory effects of Korean plants on HIV-1 activities. Phytother Res.

[10] Ahmed I, Mahmood Z, Mohammad F (1998). Screening of some Indian medicinal plants for their antimicrobial properties. J Ethnopharmacol.

[11] Mohana Krishnaswamy, Purushothaman KK (1980). Plumbagin: A study of its anticancer, antibacterial and antifungal properties. Indian J Exp Biol.

[12] Iwu MM, Jackson JE, Schuster BG (1994). Medicinal plants in the fight against leishmaniasis. Parasitol Today.

[13] Nayana SK, Shalini AI, Mamta BS (2005). A simple method for isolation of plumbagin from roots of *Plumbago rosea*. Pharm Biol.

